# Complex Relationships between Diagnostics and Survival in Chronic Lymphocytic Leukemia in Denmark, Finland, Norway, and Sweden

**DOI:** 10.3390/cancers16183229

**Published:** 2024-09-22

**Authors:** Kari Hemminki, Frantisek Zitricky, Asta Försti, Tuija Tapaninen, Akseli Hemminki, Gunnar Juliusson, Carsten Utoft Niemann

**Affiliations:** 1 Biomedical Center, Faculty of Medicine, Charles University, 323 00 Pilsen, Czech Republic; frantisel.zitricky@lfp.cuni.cz; 2 Division of Cancer Epidemiology, German Cancer Research Center (DKFZ), Im Neuenheimer Feld 580, 69120 Heidelberg, Germany; 3 Hopp Children’s Cancer Center (KiTZ), 69120 Heidelberg, Germany; a.foersti@dkfz.de; 4 Division of Pediatric Neurooncology, German Cancer Research Center (DKFZ), German Cancer Consortium (DKTK), 72076 Heidelberg, Germany; 5 Department of Clinical Pharmacology, University of Helsinki, 00014 Helsinki, Finland; 6 Individualized Drug Therapy Research Program, Faculty of Medicine, University of Helsinki, 00014 Helsinki, Finland; 7 Department of Clinical Pharmacology, HUS Diagnostic Center, Helsinki University Hospital, 00014 Helsinki, Finland; 8 Department of Hematology, Helsinki University Hospital Comprehensive Cancer Center, 00029 Helsinki, Finland; 9 Cancer Gene Therapy Group, Translational Immunology Research Program, University of Helsinki, 00014 Helsinki, Finland; akseli.hemminki@helsinki.fi; 10 Comprehensive Cancer Center, Helsinki University Hospital, 00290 Helsinki, Finland; 11 Department of Hematology, Stem Cell Center, Skåne University Hospital, Lund, Department of Laboratory Medicine, Lund University, SE-221 84 Lund, Sweden; gunnar.juliusson@med.lu.se; 12 Department of Hematology, Rigshospitalet, Copenhagen University Hospital, 2100 Copenhagen, Denmark; carsten.utoft.niemann@regionh.dk

**Keywords:** prognosis, periodic survival, treatment, targeted agents

## Abstract

**Simple Summary:**

Chronic lymphocytic leukemia (CLL) is a common leukemia characterized by an accumulation of lymphocytes in the blood and lymphoid organs. Disease presentation is highly variable as many patients do not initially require any treatment, and a watch and wait strategy remains the standard of care for up to 50% of patients. However, for those with a progressive disease, chemotherapy is the standard treatment and has improved over the years, increasing the survival of patients. We analyze here age-specific relative survival trends in CLL through 50 years up to 2020s in Denmark, Finland, Norway, and Sweden using the NORDCAN database. The large age-specific survival differences in 1972–76 almost disappeared by 2017–21. While 5-year survival in younger patients exceeded 90%, for those diagnosed at age 80–89-years, survival improved later, reaching 90% in Denmark and less in the other countries. Survival in Denmark is probably among the best in the world, which could be achieved by boosting survival even among the oldest patients. Most Nordic survival rates were better than those in the USA.

**Abstract:**

Background: Chronic lymphocytic leukemia (CLL) is a common hematological malignancy with highly variable clinical presentation. Many patients never require any treatment but for the others, chemotherapy, immunochemotherapy, and newer targeted therapies have changed the treatment landscape. Diagnostic age influences the applied treatment, and we thus wanted to analyze age-specific survival trends through 50 years up to 2020s. Methods: We used 1- and 5-year relative survival from the NORDCAN database, with data from Denmark (DK), Finland (FI), Norway (NO), and Sweden (SE). Because of the variable presentation of CLL, we also considered incidence and mortality trends. For comparison, US SEER data were used. Results: The large age-specific survival differences in 1972–76 almost disappeared by 2017–21. While 5-year survival in younger patients exceeded 90%, for those diagnosed at age 80–89 years, survival reached 90% in DK and SE women, 80% in NO and SE men, but only 50% in FI. DK 5-year overall survival for men was 92.4%, and for women, it was 96.3%. These survival figures were higher than age-group-specific US survival data. Conclusions: The DK data are probably global top figures for national survival which could be achieved by boosting survival even among the oldest patients. The qualification to these figures and international comparisons is that survival needs to be considered in terms of incidence, which is high in DK and NO. Low survival of the FI 80–89-year-old patients, even in the first year after diagnosis, may suggest delayed diagnosis, which should call for a closer national scrutiny.

## 1. Introduction

Chronic lymphocytic leukemia (CLL) is characterized by the accumulation of mature CD5-positive B-lymphocytes in the blood and lymphoid organs [1]. Treatment of CLL has changed over the years and the recent improvements have been based on increasing molecular understanding of the disease mechanisms [2]. Alkylating agents (chlorambucil), glucocorticoids, and nucleoside analogues were initially applied in the treatment of CLL, as in many other hematological malignancies after 1950s [1,3]. From the year 2000, fludarabine was used as a monotherapy and later in combination with cyclophosphamide [4]. Survival increased when a monoclonal antibody, rituximab, targeting B-cell surface antigen CD-20, was administered with purine analogues and alkylating agents (fludarabine–cyclophosphamide-rituximab, called ‘chemoimmunotherapy’, a later alternative was bendamustine–rituximab), which became the standard of care from about 2010. Another monoclonal antibody, alemtuzumab, binding to CD-52 and depleting B-cells, was used for high-risk patients with the chromosomal deletion at 17p (p53 locus), as they responded poorly to chemotherapy [5,6]. Since about 2015, novel inhibitors of kinases in the B-cell receptor signalling pathway, PI3Kδ and BTK; BCL2 antagonist, venetoclax; and further CD-20 inhibitors have transformed the recent management of CLL patients [1,6,7]. Age and comorbidities limit the use of chemoimmunotherapy and other therapeutic options, such as the obinutuzumab–chlorambucil regimen, and individual patient evaluation may help to choose optimal treatment [8]. However, many patients do not initially require any treatment, and a watch and wait strategy remains the standard of care for 30 to 50% of CLL [1,9]. Even intermediate-stage patients may remain long periods without treatment [1]. However, IGHV-unmutated + TP53-aberrated high-risk patients, which may account for 35–40% of patients at the time of diagnosis, may require treatment shortly after diagnosis [10].

CLL patients have an increased risk for infections, and many patients are diagnosed when evaluated for infectious problems. The role of infectious disease in CLL epidemiology has been extensively studied in Denmark [11,12,13,14]. Control of infections is an important part of the therapy, and machine learning may now assist the diagnostics [4,15,16,17]. In Sweden, bacterial infections leading to hospitalization were about 5 times more common in CLL patients than in matched controls [18]. Mortality in CLL patients with infection leading to in-patient treatment was 5 times higher during the first year compared to control CLL patients [18]. Novel medications, such as BTK inhibitors, have widened the scope of pathogens causing infections in CLL, but the risks can be mitigated by the selection of the treatment [15,19,20,21].

Survival in hematological malignancies has generally improved in economically developed countries, but large age-group differences continue to exist [22,23,24,25,26,27]. Much of the existing literature is of relatively short duration because of diagnostic changes and because reliable cancer registration is rather recent in many countries. The Nordic countries are an exception because they set up national cancer registries before any other countries [28]. Grouped data from these registries have been organized into the NORDCAN database, now maintained at the International Agency for Research on Cancer (IARC) [29,30]. It has been the source of many survival studies, also covering hematological malignancies [31,32,33]. NORDCAN recently added age-group-specific survival data, which inspired us to analyze age dependence in 1- and 5-year-relative survival and 5/1-year-conditional survival in CLL from Denmark (DK), Finland (FI), Norway (NO), and Sweden (SE) between 1972 and 2021. In the Nordic countries, chemoimmunotherapy became the standard treatment for fit patients in about 2010 [4,34,35,36]. For old and unfit patients, chlorambucil or bendamustine, with or without CD20 antibodies, have been used [8]. Towards 2015, new targeted therapies were introduced, but their possible survival impact may only be seen in the last 5-year period depending on the extent of their use [6,8,16,20,21].

We realized a priori the challenge of CLL for survival studies because the date of diagnosis is ambiguous, depending on many disease- and health-care-related, social, and random factors. NORDCAN records no diagnostic factors, such as stage, nor would they exist for the whole national patient population (except in national DK CLL register) [37]. We decided to face the challenge by careful scrutiny of incidence and (cause-specific) mortality trends parallel to the survival trends. The intrinsic problem with cause-specific mortality in a disease like CLL is that patients die of diverse causes, and the main cause in DK has been infection since about year 2000 [4].

## 2. Methods

The data originate from the NORDCAN database, which is a compilation of grouped data from the Nordic cancer registries [28,29]. The cancer registries in the Nordic countries are operating in a fairly similar way, and they have a long history of collaboration, as described [28]. Notifications to the registry are sent by the clinicians who diagnose cancers. The registries collect information of deaths from the national death registers and/or the causes of death registers [28]. Causes of death are determined by the death registrars, who may or may not have access to medical documents to support the cause of death. Thus, causes of death may be inaccurate, particularly for old persons [38]. We accessed the NORDCAN database at the IARC website in the winter 2024 (https://nordcan.iarc.fr/en/database#bloc2, accessed on 19 September 2024). CLL is defined by the International Classification of Diseases (ICD) version 10 code C91.1. Changes in diagnostic criteria for CLL over the years are discussed elsewhere [4,34].

Survival data were available from 1972 through 2021 in 10 5-year periods; 1-year relative survival was presented for each 5-year period and not annually. Data for 5-year relative survival were based on the cohort survival method for all but the last 5-year period, for which a hybrid analysis was used to combine period and cohort survival [29]. Conditional 5/1-year-relative survival indicates survival for those who survived the first year to survive another 4 years. Age-standardized relative survival was estimated using the Pohar Perme estimator [39]. Age-standardization was performed by weighting individual observations using external weights, as defined on the IARC web site [40]. National general population lifetables stratified by sex, year, and age were used in the calculation of expected survival. Death-certificate-only cases were not included. Patients 90 years or older were excluded. The relative survival estimates for age-specific cohorts were available if a minimum of 30 patients were alive at the start of the follow-up.

The temporal trends in relative survival were modelled using generalized additive models (Gaussian link function) in Bayesian framework [41]. The modelling was performed on cumulative hazard scale and included uncertainty of estimates derived from asymmetric confidence intervals (CIs) provided by NORDCAN. As a model input and for visualization purposes, the relative survival estimates for each period were assigned a time point in the middle of the respective 5-year period. Separate models were fitted for each country and metric.

Age-standardized incidence and mortality data were obtained from NORDCAN. It should be noted that mortality is disease-specific (cause of death CLL or other causes as defined by the death registrar), while, for relative survival, any death cause is considered. As pointed out above, the cause of CLL death in DK is recently most-commonly an infection [4].

Survival data from USA were accessed at the US Surveillance, Epidemiology, and End Results (SEER) website for the years 2015–19 for Whites and Hispanics (https://seer.cancer.gov/statistics-network/explorer/application.html?site=1&data_type=1&graph_type=2&compareBy=sex&chk_sex_3=3&chk_sex_2=2&rate_type=2&race=1&age_range=1&hdn_stage=101&advopt_precision=1&advopt_show_ci=on&hdn_view=0&advopt_display=2#graphArea, accessed on 10 March 2024).

## 3. Results

### 3.1. Case Numbers

Table 1 shows CLL patient numbers in age-groups in the Nordic countries 1972–2021. The total number of male patients was 13,000 in SE, 10,000 in DK, and 6000 in FI and NO. The number of female patients was 8000 for SE, 7000 for DK, and over 4000 for FI and NO. The highest numbers of male patients were found in age-group 70–74 years and female patients in age-groups 75–79 years. The lowest numbers for men and women were in the age-group below 50 years, but for women the 50–59 years age-group also had equally low case numbers. In the 2017–21 period, the estimated median age of onset was 72 years for men (70 years in NO) and 73/74 years for women (72 years in NO).

### 3.2. Relative Survival

In Supplementary Table S1, the overall 1- and 5-year-relative survival data are shown. Survival improved in all countries over the 50 years; SE dominated with best survival figures in the first periods and DK in the latest periods. In 1972–76, DK 5-year survival was the lowest, and in 2017–21, it was the highest (92.4% for men and 96.3% for women) of the Nordic countries. Up to around the year 2000, FI 5-year survival was higher than the DK one, but, in 2017–21, it was 10% or more below the DK level and showed no improvement for male survival in the past 10 years.

**Figure 1:**
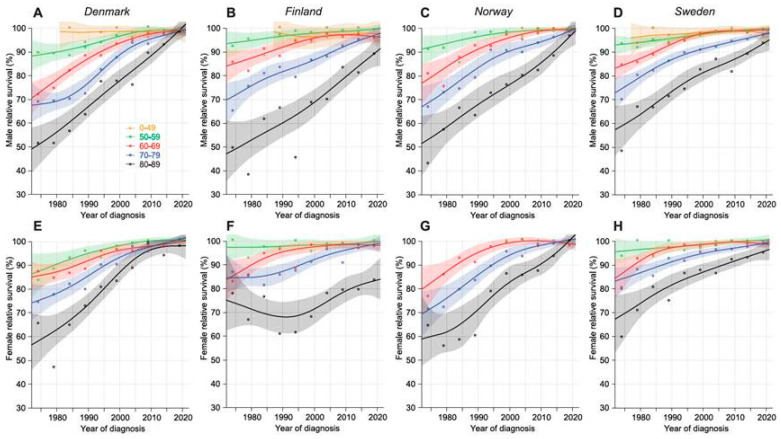
Relative 1-year survival with 95%CIs in CLL in the Nordic countries from 1972–76 to 2017–21 based on the NORDCAN database. Male data are on the top (Denmark (**A**), Finland (**B**), Norway (**C**), and Sweden (**D**)) and female data on the bottom row (Denmark (**E**), Finland (**F**), Norway (**G**), and Sweden (**H**)). Note that the curve for male patients diagnosed before age 50 years missed early periods, and for females and NO males, the complete curve was missing. For NO women also, the curve for 50–59-year-olds was missing.

In Figure 1, 1-year-relative survival is shown in age-groups through the 50-year period. Because of low case numbers, data for patients diagnosed before age 50 years were incomplete for men in the early period and completely missing for women. Survival trends were similar in each country, with a difference of about 40% units in men and 30% units in women between the age groups in 1972–76 and converging to a very small or no difference in 2017–2021. However, the FI 80–89-year-old did not catch up with their younger mates, and elderly females reached only 80% survival.

**Figure 2:**
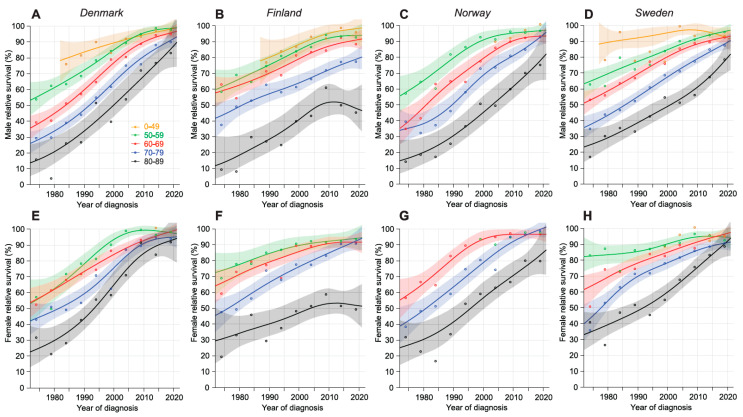
Relative 5-year survival with 95% CIs in CLL in the Nordic countries from 1972–76 to 2017–21 based on the NORDCAN database. Male data are on the top (Denmark (**A**), Finland (**B**), Norway (**C**), and Sweden (**D**)) and female data on the bottom row (Denmark (**E**), Finland (**F**), Norway (**G**), and Sweden (**H**)). Note that the curve for male patients diagnosed before age 50 years missed early periods, and for females and NO males, the complete curve was missing. For NO women also, the curve for 50–59-year-olds was missing.

Relative 5-year survival is shown in Figure 2. DK and SE female survival curves converged at or above 90%. For NO, FI women and SE men survival for patients younger than 80 years reached 90%, but for the older patients, survival was 10% units lower (40% units lower for FI women). FI 70–79-year-old men reached 80% survival, and the oldest men remained below 50% as their survival started to decline.

**Figure 3:**
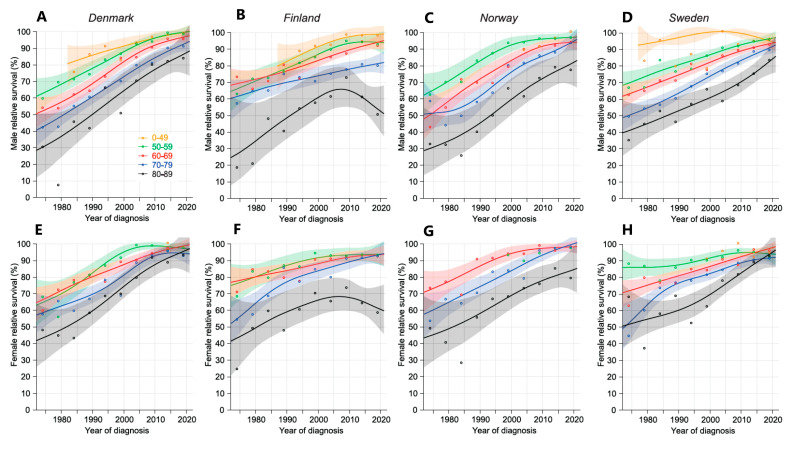
Relative 5/1-year-conditional survival in CLL with 95% CIs in the Nordic countries between 1972 and 2022. Male data are on the top (Denmark (**A**), Finland (**B**), Norway (**C**), and Sweden (**D**)) and female data on the bottom row (Denmark (**E**), Finland (**F**), Norway (**G**), and Sweden (**H**)). Note that the curve for male patients diagnosed before age 50 years missed early periods, and for females and NO males, the complete curve was missing. For NO women also, the curve for 50–59-year-olds was missing.

Conditional 5/1-year survival depicts survival for those who were alive at year 1 to survive another 4 years (Figure 3). The shapes of the curves resemble those of Figure 1, but the starting levels were shifted some 30% units lower. For DK, NO, FI women and SE men the curves for patients below age 80 years almost merged at 90–100% in 2017–21. 80–89-year-old female DK and SE patients reach 90% survival, and the other groups remained 10% units below, except for FI, whose survival turned down after year 2005 and ended at 50–60%, 30–40% units below the others.

In the US SEER database, the 5-year survival figures for CLL in 2015–19 were 88.0% for men and 89.7% for women. The age-specific data were available in three age-groups: below 50, 50–64, and 65+ years. For men, the related survival figures were 94.4, 94.0, and 83.6%; for women, they were 93.8, 95.9, and 86.5%.

### 3.3. Incidence and Mortality Rates

As diagnostic activity and procedures for CLL have changed over the years, we obtained age-standardized incidence and mortality rates from the NORDCAN database (Supplementary Figure S1). DK males showed the highest incidence, reaching a maximum of 5/100,000 in 2015, following a decline (Supplementary Figure S1A). DK female rates were also the highest, but these were only a half of the DK male rates, which was approximately the male/female rate ratio also for the other countries throughout the 50-year period. The increase in incidence rates between 1972 and 2021 was highest in NO (2.4-fold for men and 2.5-fold for women), followed by SE (1.8/2.0), DK (1.3/1.4) and FI (0.9/1.2). The incidence in 70+-year-old was an order of magnitude higher than for all patients, but the order and patterns were similar (Supplementary Figure S1C).

Mortality in CLL for the entire population showed a maximum at around 1980–90, initially followed by a slow decline and later a steeper decline (Supplementary Figure S1B). Remarkably, by 2021, all female mortality rates merged, and male rates approached each other, with NO men showing the lowest mortality. For the 70+-year-old patients, the patterns were quite similar even though the absolute rates were an order of magnitude higher (Supplementary Figure S1D).

Table 2 shows the incidence and mortality rates in years 2017–21 in three age-groups: 0–69, 70–79, and 80+ years. In all age-groups, the DK incidence rates were highest and the FI rates lowest, with a significant difference to DK (non-overlapping 95% CIs). For mortality, the country-related differences were smaller and for male rates there were no significant differences. The FI female 80+-year rate was significantly lower than the DK rate.

## 4. Discussion

We could document a positive survival development in the Nordic countries through the 50-year follow up, DK reaching 5-year relative survival in 2017–21 for men of 92.4% and for women 96.3%. Survival in NO and SE was only marginally lower but 5-year survival in FI was significantly below the other countries. Compared to the US SEER data for 2015–19 for 5-year survival of 88.0% for men and 89.7% for women, only FI data were below these figures. The UK 5-year survival up to 2023 was 86.8% for men and 87.0% for women [42]. The DK survival results are very competitive internationally, but we need to remember the caveat about high DK incidence levels [26,27]. In the European survival analysis from 2000 to 2007, DK had somewhat lower survival for CLL/small lymphocytic lymphoma than SE and NO, or the leading country Switzerland [22]. The latest Swiss data for 5-year survival of 83.8% (no sex specific data were given) are for the period 2012–2016 [26]. DK survival in those years was 90.9% for men and 94.4% for women, underlining the DK achievement. Because the median age of onset for CLL is over 70 years, the achievements would not have been possible without survival improvement in patients older than 70 years, which DK took well care of, as witnessed in Figure 1 and Figure 2 and reported in an earlier study [4].

Relative survival is a robust method in international comparisons when use of mortality rates may not be feasible because of variability in the definitions of causes of death. However, relative survival depends on the definition of the case population and is sensitive to the stage variability. A DK incidence study concluded that time-related improvements in diagnostic methods led to a relative increase in early stage CLL, similar to conclusion from NO [4,34]. The present incidence trends were in line with such observations. Incidence was highest in DK throughout the 50-year period, which was also true for the 70+-year-old population. It has also been increasing further in DK and SE but not as much as in NO. No increase took place in FI. In NO, the surge in incidence which took place starting before year 2000 has been attributed to the national introduction of immunophenotyping and to the assignment of personal doctors to each inhabitant [34]. The parallel incidence changes between sexes in each country that we observed support the notion of some general health-care-related changes. The fact that the relative survival for elderly patients in FI was below the Nordic level, but mortality was not (Table 2), may indicate that the FI elderly patients were dying relatively frequently of non-CLL-related causes. The reason for this could be that the diagnostics of CLL in FI included more advanced CLL cases (because of lower incidence) with higher proportions of comorbidities. However, the weakness of mortality data for CLL was illustrated by the DK study showing the diverse causes of death with infectious diseases becoming the main cause since year 2000 [4]. The bottom line is that for CLL survival assessment, relative survival appears to be the method of choice in the Nordic setting with the caveat that for ranking and longitudinal comparison of survival trends, the preceding incidence rates need to be considered.

How are the excellent survival figures among the DK elderly patients achieved? As discussed, the high incidence in CLL is likely to contribute to the favourable survival [4]. According to the DK national CLL guidelines, standard first-line treatment for patients older than 65 years includes bendamustine with rituximab [43]. For unfit patients with comorbidities, chlorambucil monotherapy or combination therapy with anti-CD20-antibodies are recommended, although a recent study found monotherapy unsatisfactory [43]. The same study and the recent literature lend support to the use of venetoclax plus obinutuzumab and ibrutinib-based therapies as additional options for frontline therapy [43]. Survival in CLL in DK benefits from inclusion of many patients in clinical trials which also recruit frail/elderly patients. Also, control of infections and appropriate supportive care (vaccinations and early antibiotics) are important for frail and old CLL patients [4]. Mortality in infectious disease would depress even 1-year survival, which was well controlled in all countries but FI (Supplementary Table S1): DK female 1-year relative survival in 2017–2021 was 100.1%. As additional DK resources, the CLL guidelines (https://www.lymphoma.dk/wp-content/uploads/2022/04/DLG_Kronisk-lymfatisk-leukaemi-CLL_v.-2.1_AdmGodk310322.pdf, accessed on 1 February 2024) are updated biannually, and clinical data on all patients have been collected to the national CLL registry since 2008 as a rich resource for clinical follow-up studies [43]. This resource also recently demonstrated that the mortality in CLL patients was not negatively impacted during the COVID pandemic, despite the increased mortality in CLL patients infected by COVID [44]. Unfortunately, such detailed data are not collected by the cancer registries.

Why FI relative survival trends started to lag below those for the other countries is not known, and FI studies on CLL cite no published FI CLL survival data [36,45]. Overall survival in FI was initially at the level of the other countries, but 5-year survival started to lag behind the others from 2002 to 2006 onwards. As the FI incidence did not change, we could assume that the stage distribution did not change. The weaknesses were revealed in age-specific survival for age-groups older than 70 years. Even 1-year survival for the 80–89-year-old women did not improve, which suggests that the patients were diagnosed late and died in disease complications/infections or comorbidities. Curiously, conditional survival for the oldest FI patients showed increasing mortality after around 2010, and this group of patients accounted for almost a quarter of all patients. The fact the FI survival for patients younger than 70 years was not essentially lower than that for the other countries suggested that the early chemotherapy and later immunochemotherapy were properly delivered to patients younger than 70 years. Also, the current FI guidelines for CLL are at the international level (https://hematology.fi/hoito-ohjeet/hoito-ohje-1/lymfoproliferatiiviset-taudit/kll/, accessed on 21 February 2024).

We have recently published Nordic survival data on other hematological malignancies, multiple myeloma and acute myeloid leukemia [46,47]. The results resemble the present ones about the recent backsliding of the FI survival figures compared to the other Nordic countries, particularly among old patients. Thus, the present results may conform to the pattern of slow progress in FI hematology or cancer care in general. Economy is most likely playing a role. In 1990 the Nordic countries funded health care with approximately the same sum/capita but since then FI has fallen behind the others with ever increasing gap towards year 2020 (OECD (2023), Health spending (indicator). doi: 10.1787/8643de7e-en.). With financial shortages, the level of treatment for the oldest patients may be compromised. Also in FI no national cancer policy has been instituted, opposites to the other Nordic countries [48]. The availability of novel medication may be part of the problem (https://cancerio.org/wp-content/uploads/2022/03/Cancer-Immunotherapies-in-Finland.pdf, accessed on 14 September 2024). The Nordic countries generally follow the recommendations and the scientific evaluations of the European Medicines Agency (EMA) on the release of new drugs. However, instead of a national policy of the other Nordic countries, FI applies a two-phase acceptance policy at the national and regional level, which may delay use of novel drugs.

The obvious limitation of the study is that no diagnostic or clinical data are available for the present study spanning 50 years. However, stage data are available in the DK national CLL register from 2008 onwards showing survival association with stage [37]. However, comparable stage data are lacking from the other countries. The unique power of the present study is the data available from high-level cancer registries with a long history of collaboration and thus with uniform diagnostic principles [28]. Also, the possibility to compare data from different countries gives an opportunity to assess the performance of health care systems in each and to propose improvements.

## 5. Conclusions

Our analysis of relative survival in CLL within the four Nordic countries during the 50-year period could document a consistent survival improvement across the four countries, approaching or exceeding 90% relative survival in the last time period. The largest improvement in relative survival was seen in DK from 1990 onwards reaching a national 5-year relative survival of 92.4% for men and 96.3% for women, which may be an international top achievement and should guide other countries in improving survival in CLL. This has probably been accomplished through centralized care (only eight hospitals covering all hematology care for a background population of 6 million inhabitants), good adherence to the national CLL guidelines with updated treatment and care recommendations, patient registration for follow-up purposes, including recommendation of vaccination against pneumococci and influenza, and easy access to a tax-paid public healthcare system. However, as the incidence in CLL in DK (and NO) has been high and increasing, it is likely that the positive survival at least in part also reflects a larger share of early diagnosed, relatively low-risk patients in these countries. We documented backsliding FI survival trends for the elderly patients, while survival in younger patients kept up with their Nordic mates. The consistently improved relative survival over 50 years for patients diagnosed with CLL in four countries with easy access to free public health care exemplifies paths towards nationwide improved care for such frail patients with malignancies and impaired immunity.

## Figures and Tables

**Table 1:** CLL patient numbers in age-groups (years) in the Nordic countries 1972–2021 (NORDCAN).

Population	Number	0–49	50–54	55–59	60–64	65–69	70–74	75–79	80–84	85+
Male										
Denmark	10096	433	531	860	1233	1650	1883	1591	1150	765
Finland	6182	319	339	520	785	1013	1136	892	670	508
Norway	6060	283	316	538	813	927	1005	940	696	532
Sweden	13074	497	640	1029	1543	2106	2425	2243	1599	992
Female										
Denmark	6721	226	257	483	698	929	1125	1174	982	847
Finland	4554	185	171	295	501	604	724	801	659	614
Norway	4178	176	172	277	445	566	665	663	601	661
Sweden	8053	280	303	518	815	1133	1427	1462	1221	894

**Table 2:** Incidence and mortality of CLL in Nordic countries in specific age groups during years 2017–2021 with case numbers, age-adjusted rates (ASRworld) per 100 000 and 95% CIs.

	**Age-Specific Incidence: Men**		
Age group	0–69	70–79	80+
Country	N ASR [95% CI]	N ASR [95% CI]	N ASR [95% CI]
Denmark	609 2.8 [2.6 3.0]	580 43.2 [39.7 47.0]	286 53.2 [47.1 59.9]
Finland	414 1.9 [1.7 2.1]	344 27.1 [24.3 30.2]	220 39.7 [34.6 45.4]
Norway	534 2.8 [2.5 3.0]	365 35.0 [31.5 38.8]	202 45.5 [39.4 52.3]
Sweden	764 2.1 [1.9 2.2]	716 30.1 [27.9 32.4]	423 39.8 [36.1 43.8]
	**Age-Specific Incidence: Women**		
Age group	0–69	70–79	80+
Country	N ASR [95% CI]	N ASR [95% CI]	N ASR [95% CI]
Denmark	342 1.5 [1.4 1.7]	325 21.8 [19.4 24.4]	228 28.0 [24.5 31.9]
Finland	221 1.0 [0.8 1.1]	209 13.8 [12.0 15.9]	156 15.8 [13.4 18.5]
Norway	286 1.5 [1.4 1.7]	202 18.1 [15.7 20.9]	166 23.9 [20.4 27.9]
Sweden	371 1.0 [0.9 1.1]	444 17.4 [15.8 19.1]	299 19.0 [16.9 21.3]
	**Age Specific Mortality: Men**		
Age group	0–69	70–79	80+
Country	N ASR [95% CI]	N ASR [95% CI]	N ASR [95% CI]
Denmark	34 0.15 [0.10 0.23]	106 7.5 [6.2 9.2]	155 31.5 [26.7 37.0]
Finland	54 0.24 [0.18 0.33]	89 6.8 [5.5 8.4]	170 32.4 [27.7 37.7]
Norway	25 0.13 [0.08 0.21]	52 4.8 [3.6 6.4]	113 26.5 [21.8 31.9]
Sweden	70 0.19 [0.15 0.25]	182 7.3 [6.2 8.5]	326 32.0 [28.6 35.7]
	**Age-Specific Mortality: Women**		
Age group	0–69	70–79	80+
Country	N ASR [95% CI]	N ASR [95% CI]	N ASR [95% CI]
Denmark	15 0.07 [0.04 0.13]	37 2.3 [1.6 3.2]	156 19.2 [16.3 22.5]
Finland	12 0.04 [0.02 0.12]	37 2.5 [1.7 3.4]	135 13.5 [11.3 16.0]
Norway	11 0.06 [0.03 0.14]	23 2.0 [1.3 3.1]	98 13.3 [10.8 16.3]
Sweden	26 0.07 [0.04 0.11]	52 1.9 [1.4 2.6]	262 16.0 [14.1 18.1]

## Data Availability

Publicly available data were used from the NORDCAN database.

## Supplementary Materials

The following supporting information can be downloaded at: https://www.mdpi.com/article/10.3390/cancers16183229/s1, Supplementary Figure S1. Age-standardized (world) incidence (A,C) and mortality (B,D) trends for CLL in the Nordic countries from 1972 to 2021 based on the NORDCAN data. Panels A and B show rates for the whole population, and panels C and D show rates for 70+ old patients. Male curves are solid and female curves broken. The curves were smoothened with setting 0.2. Table S1. Age-standardized 1-year and 5-year relative survival (Pohar Perme estimates with 95% CI bounds) in chronic lymphatic leukemia in Nordic countries (1972–2021). Asterisk indicates significant increase in relative survival between the marked and the next period (non-overlapping 95% CIs). Underlining shows the highest sex-specific survival in that period.

## Abbreviations

CLL	chronic lymphocytic leukemia
DK	Denmark
FI	Finland
NO	Norway
SE	Sweden
SEER	Surveillance: Epidemiology and End Results
