# Supplementary material for: Complex Relationships between Diagnostics and Survival in Chronic Lymphocytic Leukemia in Denmark, Finland, Norway, and Sweden
Source: Cancers (Basel). 2024 Sep 22;16(18):3229. doi: 10.3390/cancers16183229 (PMC11429559; doi:10.3390/cancers16183229)
Supplement: Supplementary file 1 [file cancers-16-03229-s001.zip › cancers-3213690-supplementary.pdf]

Supplementary Table S1 Age-standardized 1-year and 5-year relative survival (Pohar Perme estimates with 95% CI bounds) in chronic lymphatic leukemia in Nordic countries (1972-2021). Asterisk indicates significant increase in relative survival between the marked and the next period (non-overlapping 95% CIs). Underlining shows the highest sex-specific survival in that period.

	Male 1-year relative survival				Female 1-year relative survival			
**Period**	**Denmark**	**Finland**	**Norway**	**Sweden**	**Denmark**	**Finland**	**Norway**	**Sweden**
1972-1976	72.7[68.5-77.0]	76.0[71.3-81.1]	74.9[70.3-79.8]	77.9[74.6-81.4]	80.1[75.2-85.2]	88.5[84.6-92.7]	77.6[71.6-84.0]	81.0[76.8-85.4]*
1977-1981	73.7[70.1-77.5]	77.1[73.1-81.4]	76.7[72.5-81.2]	83.5[80.8-86.3]	79.7[76.1-83.4]	85.3[81.6-89.1]	80.5[76.1-85.1]	90.5[87.9-93.1]
1982-1986	78.3[75.4-81.3]	84.2[80.6-88.0]	80.9[76.5-85.5]	86.2[84.0-88.4]	85.0[81.9-88.2]	88.5[85.1-92.1]	81.9[76.3-87.9]	89.8[87.1-92.5]
1987-1991	81.5[78.8-84.4]*	86.8[83.2-90.5]	85.7[82.0-89.5]	88.8[86.5-91.2]	85.9[82.8-89.1]	88.9[85.8-92.0]	84.2[79.6-89.1]*	93.6[91.6-95.7]
1992-1996	87.4[84.9-90.0]	83.7[80.5-87.0]	90.5[87.4-93.8]	90.7[88.7-92.7]	91.6[89.0-94.2]	87.8[84.8-90.8]	92.8[89.9-95.9]	94.7[93.0-96.5]
1997-2001	91.0[88.9-93.1]	89.3[86.4-92.3]	93.0[90.4-95.6]	93.5[91.9-95.1]	93.6[91.6-95.7]	91.7[89.1-94.4]	96.7[94.7-98.8]	95.8[94.2-97.4]
2002-2006	92.5[90.7-94.4]	90.9[88.5-93.4]	92.5[90.2-94.8]	95.3[93.9-96.6]	96.7[95.2-98.3]	95.0[92.9-97.1]	96.4[94.7-98.1]	96.4[95.1-97.7]
2007-2011	95.7[94.1-97.3]	93.4[91.2-95.7]	95.1[93.3-96.9]	95.4[94.2-96.5]	98.5[97.3-99.8]	93.9[91.8-96.1]	97.4[95.9-98.9]	98.0[97.0-99.1]
2012-2016	97.5[96.3-98.7]	95.1[93.4-96.7]	97.1[95.7-98.5]	96.3[95.2-97.4]	98.4[97.2-99.6]	95.5[93.9-97.2]	98.3[96.5-100.0]	98.1[97.1-99.1]
2017-2021	98.7[97.5-99.8]	95.9[94.3-97.5]	98.9[97.8-100.0]	97.8[96.8-98.8]	100.1[99.3-101.0]	96.7[95.1-98.2]	99.8[98.8-100.9]	98.8[98.0-99.7]

	Male 5-year relative survival				Female 5-year relative survival			
**Period**	**Denmark**	**Finland**	**Norway**	**Sweden**	**Denmark**	**Finland**	**Norway**	**Sweden**
1972-1976	36.8[31.8-42.5]	46.8[41.1-53.3]	41.7[35.8-48.4]	47.1[42.5-52.2]	46.5[39.8-54.3]	56.5[50.7-63.1]	51.9[44.6-60.4]	52.5[46.7-58.9]*
1977-1981	38.3[34.1-43.0]*	50.3[45.0-56.2]	42.9[37.6-48.9]	52.2[48.1-56.6]*	51.8[46.5-57.8]	61.1[55.8-66.9]	57.5[51.0-64.7]	66.4[62.1-70.9]
1982-1986	48.9[44.7-53.5]	57.9[52.3-64.1]	49.6[43.6-56.4]	61.0[57.5-64.6]	59.3[54.4-64.6]	67.1[61.3-73.5]	57.7[50.9-65.3]	65.9[61.2-70.9]
1987-1991	53.6[49.6-58.0]*	63.5[57.6-69.9]	55.8[49.8-62.6]	59.8[55.7-64.1]	63.7[58.7-69.2]	70.9[65.5-76.7]	68.6[62.4-75.4]	74.4[69.9-79.1]
1992-1996	65.2[61.0-69.8]	63.0[57.9-68.5]	64.3[58.7-70.4]*	67.7[64.1-71.4]	72.4[67.7-77.4]	69.5[64.3-75.1]*	80.0[74.4-86.1]	76.0[72.4-79.9]
1997-2001	69.4[65.5-73.6]*	70.4[65.6-75.6]	76.5[71.4-82.0]	72.1[68.8-75.5]*	77.7[73.9-81.7]*	80.9[76.4-85.7]	85.6[81.0-90.4]	79.0[75.6-82.6]
2002-2006	77.8[74.2-81.6]	74.1[69.9-78.7]	78.3[74.1-82.7]	78.6[76.0-81.3]	86.9[83.4-90.7]*	81.6[77.4-85.9]	82.3[77.9-87.0]*	85.0[82.1-88.0]
2007-2011	84.1[80.7-87.6]*	79.9[75.9-84.1]	84.1[80.5-88.0]	81.5[79.0-84.1]	93.8[90.9-96.8]	83.0[78.8-87.3]	91.2[87.5-95.0]	88.4[85.8-91.1]
2012-2016	90.9[88.2-93.7]	82.5[79.4-85.9]	88.0[84.9-91.2]	86.1[83.8-88.5]	94.3[91.6-97.1]	85.0[81.0-89.1]	94.0[90.9-97.3]	91.4[88.8-94.0]
2017-2021	92.4[89.7-95.2]	80.4[77.2-83.6]	91.9[89.0-94.8]	89.6[87.3-91.9]	96.3[93.6-99.0]	86.4[83.2-89.7]	95.0[92.1-98.1]	92.5[90.0-95.0]

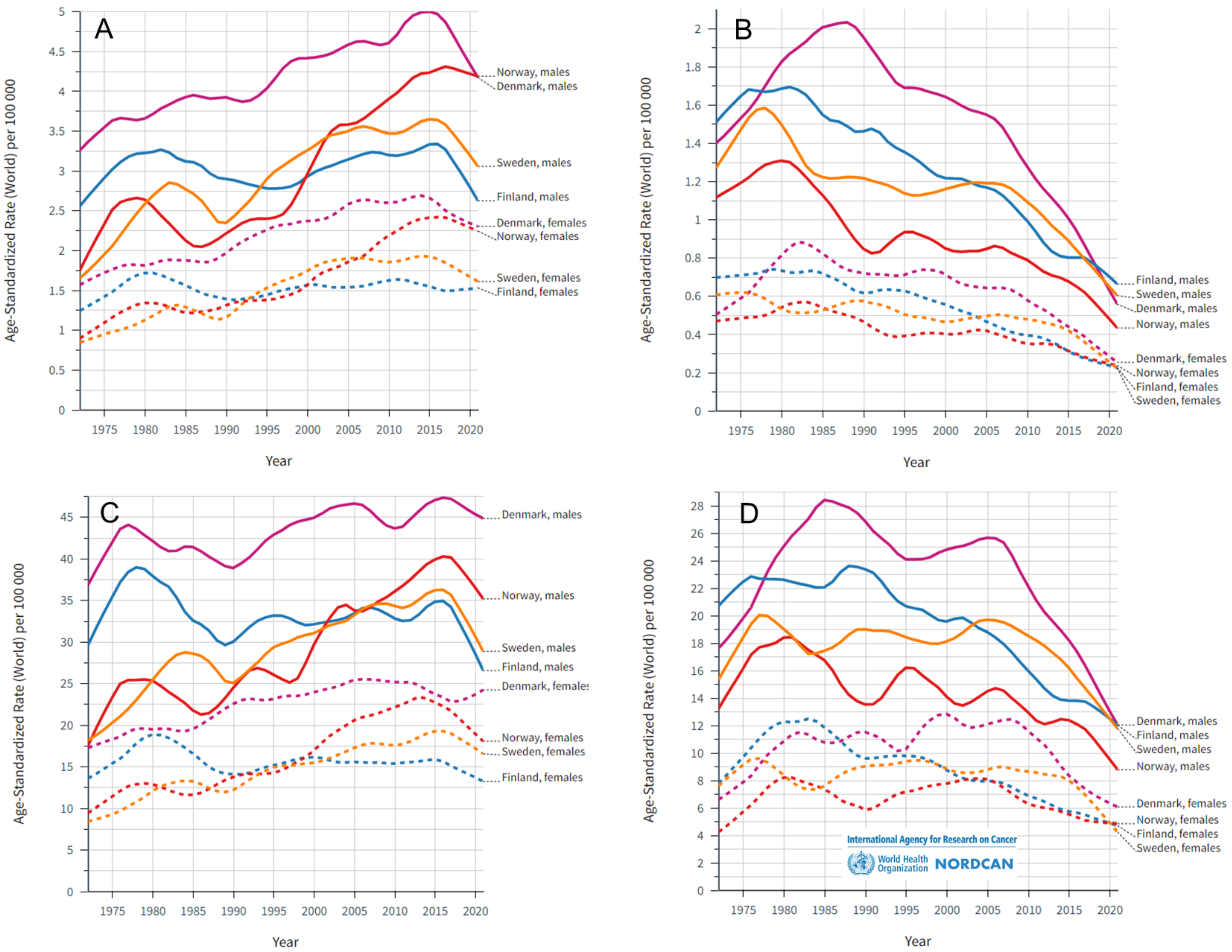

Supplementary Figure S1
